# The Manual Delivery of the Placenta

**Published:** 1849-11

**Authors:** 


*The Manual Delivery of the Placenta.*—By JOSEPH PARRISH, M. D. Editor of the New Jersey Medical Reporter.

There appeared in one of the recent numbers of the Buffalo Medical Journal, some observations on the manual delivery of the placenta, exhibiting the practice of the distinguished editor of that periodical, in this respect, which were particularly gratifying to me, as a corroboration of the practice pursued by myself, and it is believed, by a number of others; though to deliver the secundines immediately after the expulsion of the child, is not in conformity with the teachings of the schools. To wait twenty minutes, or half an hour, for the natural powers of the uterus to complete this last effort of labor, is the generally adopted practice; it has so been taught by the most eminent men, and on that account is worthy of great respect, and ought not to be changed without sufficient evidence to warrant a different course. About one hundred and thirty obstetric cases have come under my care within the past year, and the practice has been adopted in each of them, without any untoward result being apparent. Immediately after the chord is separated, and the child removed, the left hand is applied over the fundus of the uterus externally, and pressure continued for several minutes, while slight traction is made upon the chord by the right hand. If by this manœuvre, the attachments do not readily yield, the right hand is at once inserted into the vagina, or uterus, as the case may require, and the edge of the placenta hooked by the index finger, or the whole viscus embraced, and dislodged from the uterus; at the same time the nurse is directed to make friction over the lower part of the abdomen. Is it not reasonable to conclude that alarming uterine hemorrhage may be prevented by this practice, as the womb is made to contract speedily, and empty itself of its contents, by thus abruptly shutting up the mouths of the vessels which communicate between the maternal and fœtal circulation? If the placenta is allowed to remain, separated as it generally is, from the fundus-uteri, and embraced by the cervix, there is a space left in the cavity of the womb, which may be speedily filled with blood from the ruptured vessels at the fundus, and the woman suffer exhaustion from loss of the vital fluid, while the accoucheur is patiently waiting for the operation of nature. The fear of after pains is also materially lessened, as they mainly depend upon the presence of coagulated blood in the uterus, which may be more readily and completely removed, by keeping up the tonic contractions of the womb until all its contents are evacuated. The child is expelled by a powerful muscular effort; and when thrown out from the womb, the stimulus of its presence being removed, and the uterus not sufficiently contracted, it is necessary to supply an artificial stimulus by the hand, externally, and if need be, by contact with the internal surface, in order to complete the uterine action, and save the woman the suffering of after pains for several successive days, as well as relieve her from the risk of immediate hemorrhage. These suggestions are made after a fair trial of the practice, and are communicated to the profession for consideration.

After the birth of the child, not more than five minutes need be allowed, in ordinary cases, for the completion of the whole process—the placenta being removed, and the uterus brought down into the pelvic basin, without the presence within its cavity, of any offending body, to act as an irritant to its fibres, and cause the patient to suffer under their continued contraction. —*New Jersey Medical Reporter.*

